# Saturated fatty acids in human visceral adipose tissue are associated with increased 11- β-hydroxysteroid-dehydrogenase type 1 expression

**DOI:** 10.1186/s12944-015-0042-1

**Published:** 2015-05-02

**Authors:** Paul Petrus, Fredrik Rosqvist, David Edholm, Niklas Mejhert, Peter Arner, Ingrid Dahlman, Mikael Rydén, Magnus Sundbom, Ulf Risérus

**Affiliations:** Department of Medicine, Karolinska Institutet, Karolinska University Hospital, Huddinge, Stockholm Sweden; Clinical Nutrition and Metabolism, Department of Public Health and Caring Sciences, Uppsala University, Uppsala, Sweden; Department of Surgical Sciences, Uppsala University Hospital, Uppsala University, Uppsala, Sweden

**Keywords:** 11-β-hsd1, Saturated fatty acids, Gene expression, Adipose tissue, Visceral fat, Cortisol, Diet

## Abstract

**Background:**

Visceral fat accumulation is associated with metabolic disease. It is therefore relevant to study factors that regulate adipose tissue distribution. Recent data shows that overeating saturated fatty acids promotes greater visceral fat storage than overeating unsaturated fatty acids. Visceral adiposity is observed in states of hypercortisolism, and the enzyme 11-β-hydroxysteroid-dehydrogenase type 1 (11β-hsd1) is a major regulator of cortisol activity by converting inactive cortisone to cortisol in adipose tissue. We hypothesized that tissue fatty acid composition regulates body fat distribution through local effects on the expression of 11β-hsd1 and its corresponding gene (*HSD11B1)* resulting in altered cortisol activity.

**Findings:**

Visceral- and subcutaneous adipose tissue biopsies were collected during Roux-en-Y gastric bypass surgery from 45 obese women (BMI; 41 ± 4 kg/m^2^). The fatty acid composition of each biopsy was measured and correlated to the mRNA levels of *HSD11B1*. 11β-hsd1 protein levels were determined in a subgroup (n = 12) by western blot analysis. Our main finding was that tissue saturated fatty acids (e.g. palmitate) were associated with increased 11β-hsd1 gene- and protein-expression in visceral but not subcutaneous adipose tissue.

**Conclusions:**

The present study proposes a link between *HSD11B1* and saturated fatty acids in visceral, but not subcutaneous adipose tissue. Nutritional regulation of visceral fat mass through *HSD11B1* is of interest for the modulation of metabolic risk and warrants further investigation.

## Background

Increased visceral adipose tissue (VAT) mass is strongly associated with metabolic disorders whereas subcutaneous adipose tissue (SAT) expansion is regarded as less detrimental [1]. Body fat distribution is influenced by age, ethnicity and gender [2,3], and is under hormonal influence by e.g. cortisol and other steroid hormones [4]. However, body fat distribution may also be regulated by modifiable lifestyle factors such as diet. Recently, dietary fatty acid (FA) quality was shown to influence body composition and body fat distribution during hyper- and iso-caloric conditions in humans [5,6].

Cortisol has a major impact on body fat accumulation. VAT has a higher concentration of glucocorticoid receptors than SAT [4], and lipoprotein lipase activity is increased by cortisol with depot specific differences [7]. The local concentrations of cortisol are regulated by the enzyme 11-β-hydroxysteroid-dehydrogenase type 1 (11β-hsd1) encoded by *HSD11B1*, which in humans converts inactive cortisone into active cortisol. Recent data obtained in murine models, suggest that increased tissue production of cortisol by 11β-hsd1 is critical for developing the characteristic phenotype linked to conditions of hypercortisolism, e.g. VAT accumulation and insulin resistance [8]. Mice lacking *HSD11B1* display decreased VAT mass [9] whereas reciprocal effects are observed in mice over-expressing *HSD11B1* [10]. In humans, inhibition of 11β-hsd1 activity resulted in reduced VAT and liver fat content compared to placebo [11].

Interestingly, via as of yet unclear mechanisms, FAs that influence visceral fat accumulation in humans [6] have also been shown to regulate the expression of *HSD11B1* in rat VAT [12]. Thus, polyunsaturated FAs (PUFAs) inhibited, whereas saturated FAs (SFAs) increased *HSD11B1* expression in adipose tissue, which could link the differential effects of these FAs on VAT accumulation in humans.

As cortisol is a known regulator of body fat distribution and body composition, we hypothesized that FA composition in VAT influences *HSD11B1* gene and protein expression which could in turn influence VAT accumulation. Accordingly, we investigated the associations between saturated and unsaturated FA proportions and *HSD11B1*/11β-hsd1 expression in VAT and SAT collected from obese subjects.

## Material and methods

### Subjects

Forty-five obese women without diabetes that underwent a laparoscopic Roux-en-Y gastric bypass surgery at Uppsala University Hospital were recruited. Diabetes was defined as previous diagnosis or glucose lowering treatment, or a fasting plasma glucose ≥7.0 mmol/l or ≥11.1 mmol/l 2 h after an oral glucose tolerance test. According to local guidelines, patients were assigned a low calorie diet (LCD, maximum 1200 kcal/day), during one month prior to surgery. Written informed consent was obtained from all subjects. The study was approved by the regional Ethical board in Uppsala.

### Adipose tissue biopsies

During surgery, we collected biopsies from two different adipose tissue depots; SAT was collected from the upper left side of the abdomen, whereas VAT was collected from the omentum. Biopsies were washed in isotonic saline solution, placed in Eppendorf tubes and snap frozen using dry ice and ethanol.

### Fatty acid composition measurement with gas chromatography

FA composition (individual saturated, monounsaturated and polyunsaturated FAs) was measured in VAT and SAT biopsies using gas–liquid chromatography [5,13]. FAs were expressed as percentages of the total FAs analysed.

### RNA extraction, cDNA synthesis and qRT-PCR

Total RNA was extracted from all subjects using miRNeasy Mini kit (Qiagen, Hilden Germany). RNA concentration and purity was measured spectrofotometrically using a Nanodrop ND-2000 Spectrophotometer (Thermo Fischer Scientific, MA USA). High RNA quality was confirmed in 16 randomly chosen samples using Agilent R6K ScreenTape System (Agilent Technologies, Waldbronn Germany). cDNA was synthesized using iScript™ cDNA Synthesis Kit (Bio-Rad Laboratories, CA USA). The reverse transcription incubation was performed in a PTC-100R Thermal Cycler (Bio-Rad Laboratories). Relative gene-expression levels were calculated using a standard curve. The qRT-PCR was performed with iCycler IQ™ (Bio-Rad Laboratories, CA USA). TaqMan® 2x universal PCR master mix (Applied Biosystems, CA USA) and predesigned TaqMan® assay (Applied Biosystem, CA USA) targeting *HSD11B1* and the housekeeping genes *LRP10* and *18S* were used according to the manufacturer’s instructions.

### Protein extraction and Western blot

The difference in *HSD11B1* expression between SAT and VAT were calculated for each individual and a subgroup including the 12 subjects with the largest difference (SAT > VAT) were selected for protein measurements. Adipose tissues (99–101 mg) were homogenized with IKA® T10 basic ULTRA-TURRAX ( IKA®-Werke GmbH & Co. KG, Staufen Germany), protein concentration determined and western blot performed as previously described [14]. Primary polyclonal goat IgG α-11β-hsd1- (R&D systems®, MN USA) and secondary igG α-goat (Sigma Aldrich) antibodies were used.

### Statistical analysis

All statistical analysis was performed using IBM SPSS Statistics 22.0. Significant differences between two groups were tested with students´ t-test. Correlations were performed using Spearman’s correlation analysis.

## Findings

Patients (n = 45) had a mean age of 40 ± 10 years and a mean BMI of 41 ± 4 kg/m^2^. The subgroup for protein analyses consisted of 12 patients with a mean age of 34 ± 8 years and a mean BMI of 42 ± 3 kg/m^2^.

### *HSD11B1* expression in SAT and VAT

**Figure 1:**
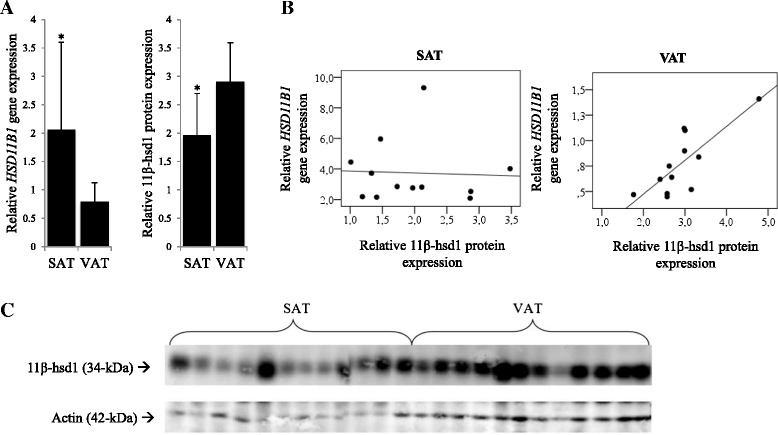
*HSD11B1* gene and 11β-hsd1 protein expression in adipose tissue depots. **A**. The gene expression of *HSD11B1* differed significantly between SAT and VAT with the higher gene expression in SAT whereas the protein expression was higher in VAT. Data are presented as means ± SD (*, differ from VAT, P < 0.001). **B**. Correlations between *HSD11B1* mRNA and 11β-hsd1 protein expression. Correlations between *HSD11B1* mRNA expression and 11β-hsd1 protein expression are illustrated in scatterplots. A statistically significant correlation is found only in the VAT (rho = 0.78 and P = 0.010). **C**. 11β-hsd1 and actin detection. Bands were detected at 34-kDa and 42-kDa when using antibodies targeting 11β-hsd1 and actin.

While gene expression data indicated higher *HSD11B1* mRNA expression in SAT than in VAT, reciprocal findings were obtained at the protein level (Figure 1A). A statistically significant correlation was found between the gene- and protein expression only in VAT of the analysed sub-group (n = 12) (Figure 1B).

### Associations between tissue fatty acids and *HSD11B1*

**Figure 2:**
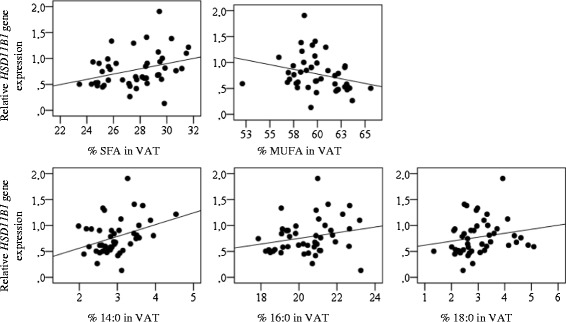
Associations between *HSD11B1* expression and individual fatty acids in VAT. Correlations between *HSD11B1* expression and FA-composition in the VAT are illustrated in scatterplots. Total SFA (rho = 0.39 and P = 0.006) and MUFA (rho = −0.38 and P = 0.007) together with the individual SFAs 14:0 (rho = 0.34 and P = 0.025) 16:0 (rho = 0.31 and P = 0.039) and 18:0 (rho = 0.30 and P = 0.049) correlated with *HSD11B1* expression.

The association between FA composition and gene expression was tested in SAT and VAT. The saturated FAs 14:0, 16:0 and18:0 correlated significantly with *HSD11B1* expression in VAT. Additionally, total VAT SFA were positively correlated whereas total VAT monounsaturated FAs (MUFAs) were inversely correlated with *HSD11B1* expression (Figure 2).

## Discussion

Herein we investigated the associations between adipose tissue FA composition and *HSD11B1* expression in humans. Our new findings suggest a potential distinct role of saturated and unsaturated FAs in regulating *HSD11B1* in human VAT. We found an association between total as well as with several long-chain SFAs (e.g. palmitate) and *HSD11B1* expression in VAT, whereas the opposite association was observed for total MUFAs. The association between FAs and *HSD11B1* expression were not present in SAT. The reason for this is unknown but could be explained by the results from the western blot suggesting that the gene and corresponding protein levels in SAT are poorly correlated. These discrepancies may be due to variation in post-transcriptional/-translational regulation of the enzyme between the depots. However, it should also be considered that the subgroup that was selected for protein measurement may not be representative of the study population. The relationship between SFAs and *HSD11B1* expression is in agreement with previous findings in rats [12] and indicates a possible mechanism influencing fat distribution. Prior studies in humans have shown that dietary fat quality influences body composition and body fat distribution with unfavourable effects from a diet rich in SFAs (palmitate) when compared to a PUFA diet [5,6,15]. The lack of inverse association between PUFAs and *HSD11B1* in VAT may be partly explained by a potentially higher beta-oxidation of PUFAs compared with SFA and MUFA resulting in lower PUFA storage in AT [16]. This possibility should be considered since the fat biopsies were collected after a low-caloric diet promoting fat oxidation and inhibiting fat storage. However, protective effects from MUFAs and PUFAs might be due to a decrease in SFA content since no inverse correlations were detected between *HSD11B1* expression and PUFA content. Additionally, the effect of MUFA on *HSD11B1* expression could be due to the inverse correlation between SFA and MUFA (unpublished data) in this study population since no known comparison between SFA and MUFA enriched diets have been done with regard to *HSD11B1* expression or body fat distribution. The addition of PUFAs to the diet, without substituting other FAs, may not influence VAT mass as indicated by a post-hoc analyses [17]. Noteworthy is that the energy balance of the diet may also influence since previous dietary FA interventions have been performed under hypo-, iso- and hyper-caloric conditions [5,6,17]. A direct effect of fatty acid species on *HSD11B1* expression could be tested by stimulating *in vitro* differentiated visceral and subcutaneous adipocytes with different fatty acids. Such experiments could unfortunately not be performed due to difficulties obtaining sufficient amount of visceral adipose tissue from patients for cell culturing.

**Figure 3:**
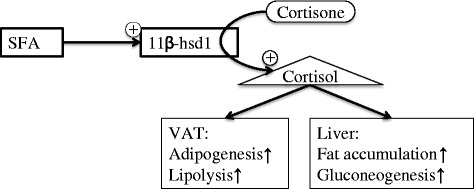
This schematic picture presents a hypothetical link between SFAs and *HSD11B1* expression. SFAs may function as signalling molecules and alter transcription of *HSD11B1*. The increased *HSD11B1* expression in VAT is associated with an increased protein expression of the enzyme which results in an elevated conversion of cortisone to cortisol. Cortisol binds to the glucocorticoid receptor (GR) which regulates transcription which results in VAT adipogenesis and increased hepatic lipid accumulation due to induced AT lipolysis. It should be noted that the suggested mechanism is simplified and a model extracted from the observed associations and ought to be confirmed before considered valid.

The molecular mechanisms explaining the associations between FA type, body composition and gene expression cannot be established from this study, however, our data together with others [6,10,12] can be extended to a theoretical model of the underlying mechanism. A schematic and simplified overview of the mechanisms is illustrated in Figure 3. Noteworthy is that the response to cortisol stimulation varies between AT depots, probably due to variations in the transcription co-factor GC-dependent gene LIM domain only 3 (LMO3) [18]. The signalling pathway explaining the increased *HSD11B1* expression upon SFA stimulation has not been established. However, a possible signalling pathway is through toll-like receptor 4. This receptor also binds TNFα, a stimulator of *HSD11B1* expression [19].

Strengths of the study include the relatively large sample size, assessment of fatty acid composition together with mRNA of *HSD11B1* and protein analyses of its coding enzyme in two key abdominal adipose tissue depots. The consistent association between several major SFAs (14:0, 16:0 and 18:0) and *HSD11B1* expression however strengthens the results and supports a potential SFA specific effect.

There are some limitations of the study. The study population may not be representative for men and for non-obese individuals since the patients were obese women. In addition, the subjects were assigned to follow a LCD which could influence with gene expression. The FA composition is not believed to be significantly influenced since the triglyceride turnover is much longer than 1 month [20], although fatty acids may be differently oxidized and thereby influencing adipose tissue FA composition [16]. Although both gene- and protein- expression was measured, we lack information on enzyme activity and cortisol levels. Due to the diurnal and inter-individual variation, a 24 h urine collection is needed to have a good estimation of cortisol levels. Lastly, this study is cross-sectional and causality cannot be established, and consequently these novel results in humans should be viewed as hypothesis generating that motivates further investigation.

## Conclusions

This is the first study in humans investigating the association between fatty acid type and *HSD11B1*expression in adipose tissue. We show distinct relationships between saturated and unsaturated FAs with regard to *HSD11B1*, in accordance with previous findings in rats. Further research is needed to establish causality, and determine whether associations with FAs in VAT reflects dietary intake or local endogenous FA metabolism, or both.

## Abbreviations

11-β-hsd1: 11-β-hydroxysteroid-dehydrogenase type 1
AT: Adipose tissue
SAT: Subcutaneous adipose tissue
VAT: Visceral adipose tissue
FA: Fatty acid
SFA: Saturated fatty acid
MUFA: Monounsaturated fatty acid
PUFA: Polyunsaturated fatty acid
